# Non-specific interactions of antibody-oligonucleotide conjugates with living cells

**DOI:** 10.1038/s41598-021-85352-w

**Published:** 2021-03-15

**Authors:** Victor Lehot, Isabelle Kuhn, Marc Nothisen, Stéphane Erb, Sergii Kolodych, Sarah Cianférani, Guilhem Chaubet, Alain Wagner

**Affiliations:** 1 Bio-Functional Chemistry (UMR 7199), LabEx Medalis, University of Strasbourg, 74 Route du Rhin, 67400 Illkirch-Graffenstaden, France; 2 BioOrganicMass Spectrometry Laboratory (LSMBO), IPHC, University of Strasbourg, 25 rue Becquerel, 67087 Strasbourg, France; 3 Syndivia SAS, ISIS, 8 allée Gaspard Monge, 67000 Strasbourg, France

**Keywords:** Drug delivery, Drug safety, Medicinal chemistry, Pharmacology, Biologics, Antibody therapy, Nucleic-acid therapeutics, Chemical biology, Chemical modification, Drug delivery, Nucleic acids, Pharmacology, Proteins, Chemical modification, DNA, Nucleic-acid therapeutics, Drug discovery

## Abstract

Antibody-Oligonucleotide Conjugates (AOCs) represent an emerging class of functionalized antibodies that have already been used in a wide variety of applications. While the impact of dye and drug conjugation on antibodies’ ability to bind their target has been extensively studied, little is known about the effect caused by the conjugation of hydrophilic and charged payloads such as oligonucleotides on the functions of an antibody. Previous observations of non-specific interactions of nucleic acids with untargeted cells prompted us to further investigate their impact on AOC binding abilities and cell selectivity. We synthesized a series of single- and double-stranded AOCs, as well as a human serum albumin-oligonucleotide conjugate, and studied their interactions with both targeted and non-targeted living cells using a time-resolved analysis of ligand binding assay. Our results indicate that conjugation of single strand oligonucleotides to proteins induce consistent non-specific interactions with cell surfaces while double strand oligonucleotides have little or no effect, depending on the preparation method.

## Introduction

Antibody-oligonucleotide conjugates (AOCs) have received increasing attention as an emerging class of chimeric biomolecules. Combining the specific binding ability of antibodies with the vast structural and functional properties of oligonucleotides (ONs), these conjugates have found a wide variety of applications as imaging, detection and therapeutic agents^1^.

All of these functions primarily require the discrimination of the targeted cell type via specific binding of the AOC to its protein target. Reaching high efficacy in therapeutic applications thus requires, among other, both a high affinity for the targeted protein at the targeted cell surface and low interactions with the other, non-targeted, cells.

In a recent work^2^, we investigated the ability of AOC constructs (termed DNA-linked ADCs) to carry and deliver a small-molecule drug into a targeted cell in a selective fashion. To our surprise, we observed that while our DNA-linked ADC (based on the anti-HER2 monoclonal antibody trastuzumab) showed a similar toxicity profile on HER2^+^ cells to classical covalent conjugates, it also showed low but unexpected toxicity on the control HER2^−^ cell line. We hypothesized that this toxicity was the result of a non-specific interaction of the conjugate with HER2^−^ cells induced by the ON linker. This puzzling observation motivated us to further investigate the impact of ON conjugation on the cell binding properties of antibodies and proteins.

ONs, because of their hydrophilic nature and multiple negative charges, constitute a singular type of payload for which a few literature reports have highlighted non-specific interactions with cell membranes. In 1995, Walker et al.^3^ described the synthesis and in vitro cellular uptake of an anti-transferrin receptor antibody-antisense ON conjugate (the antisense ON being a single-stranded DNA) and observed non-specific cell association for both control IgG-ssON conjugate and free ON. The “non-specific” interactions of non-modified^4^ and dye-labelled^5^ ONs with cell membranes have also been reported. However, despite these early warnings, the effect of ON conjugation on antibody selectivity remains understudied.

To shed light on such potentially determinant effect, we compared the interactions of various protein-ON conjugates, unconjugated proteins and free ONs with live cells using a time-resolved analysis of ligand binding assay. This technique allows the study of interactions in real-time on non-treated, live cells in culture medium, and in the presence of serum, a situation resembling in vivo conditions. Importantly, it requires no washing step that could wash off compounds before their detection^6^, revealing weak interactions with fast dissociation rates.

## Results and discussion

**Figure 1:**
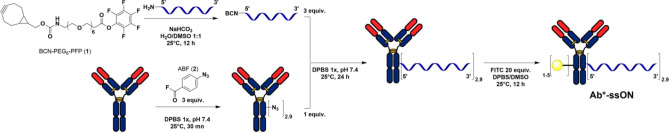
General scheme for the synthesis and labelling of Ab*-ssON conjugates.

Using a previously reported plug and play conjugation strategy^7^, we prepared several fluorescein-labelled proteins and protein-ON conjugates (see Fig. 1, 5, S1, S2 and Table S1 for syntheses and characterization of the conjugates) with Degrees of Conjugation (DoC) in line with those of our previously-described DNA-linked ADCs^2^ (i.e. comprised between 2 and 3). We then evaluated the interaction profiles of these protein-ON conjugates with two cell lines, SK-BR-3 (HER2^+^) and MDA-MB-231 (HER2^−^), using time-resolved analysis of ligand binding assay^8,9^. As an HER2 targeting antibody, we used trastuzumab and as negative controls, we used the anti-CD20 antibody rituximab and Human Serum Albumin (HSA).

In the following figures, we will report association rate constant values k_a_ (M^−1^ s^−1^), describing the rate of formation of complexes, i.e. the number of fluorescein-labelled compounds bound to cell membranes per second. Thus, high k_a_ value will account for fast binding to cell surface, while low value will account for slow to no interaction. The values of k_a_ in biological systems are typically comprised between 1.10^3^ and 1.10^7^ M^−1^ s^−1^. We chose to study this parameter to compare specific and non-specific interactions as it was previously shown to be largely insensitive to differences between binding patterns^10^.

**Figure 2:**
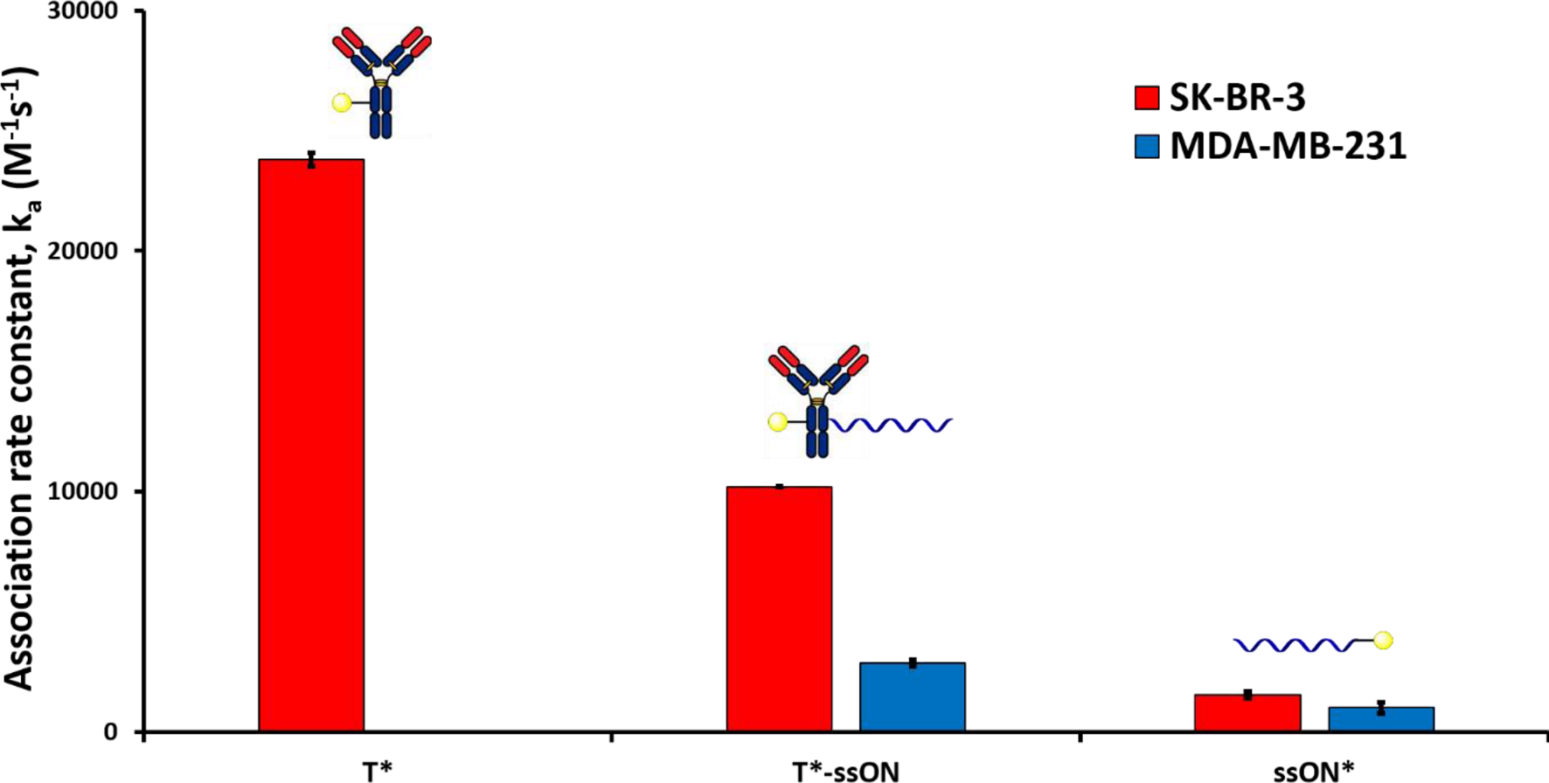
Association rate constants (k_a_) of unconjugated trastuzumab (T*), trastuzumab- single-stranded ON conjugate (T*-ssON) and free single stranded ON (ssON*) on SK-BR-3 (HER2^+^ cell line, red bars) and MDA-MB-231 (HER2^−^ cell line, blue bars) measured using the time-resolved analysis of ligand binding assay (Fig. S3, S4, and S13). The symbol * indicates fluorescein labelling. Error bars indicate the standard error of the fitted parameter k_a_.

In a first set of experiments, we compared the association rate constants of anti-HER2 antibody trastuzumab (T*), trastuzumab-37mer ssON conjugate (T*-ssON), and single-stranded 37mer ssON (ssON*), on both cell lines (Fig. 2). In order to do so, cells were seeded on a cell dish and incubated with fluorescein-labelled compounds (the symbol * indicates fluorescein labelling). The fluorescence intensity was then measured over time and normalized against the background fluorescence of the plastic support (see Fig. S3 to S16). The signal increase was used to extract the association rate constant k_a_.

Unsurprisingly, trastuzumab displayed a high selectivity profile with an association rate constant k_a_ of 23,800 M^−1^ s^−1^ on the HER2^+^ cell line, while no signal variation was detected for the HER2^−^ cell line. T*-ssON showed a deteriorated selectivity profile with a reduced k_a_ for HER2^+^ cells, but more importantly with the appearance of "non-specific" interactions with HER2^−^ cells. Stochastic conjugation with lysine residues^11,12^ for T*-ssON might account for the lower k_a_ with the HER2^+^ cell line as compared to unconjugated T*. Indeed, random conjugation of payloads, including dyes^11^ and small-molecule drugs^12,13^, to an antibody was reported to deteriorate its antigen affinity in some cases.

It is noteworthy that ssON* showed an HER2-independant interaction with both cell lines, as evidenced by low but significant values of k_a_ on both SK-BR-3 and MDA-MB-231 cell lines. The order of magnitude of these interactions falls in the same range as that of T*-ssON on HER2^−^ cells, advocating for the fact that the latter was the result of ON-cell interactions.

**Figure 3:**
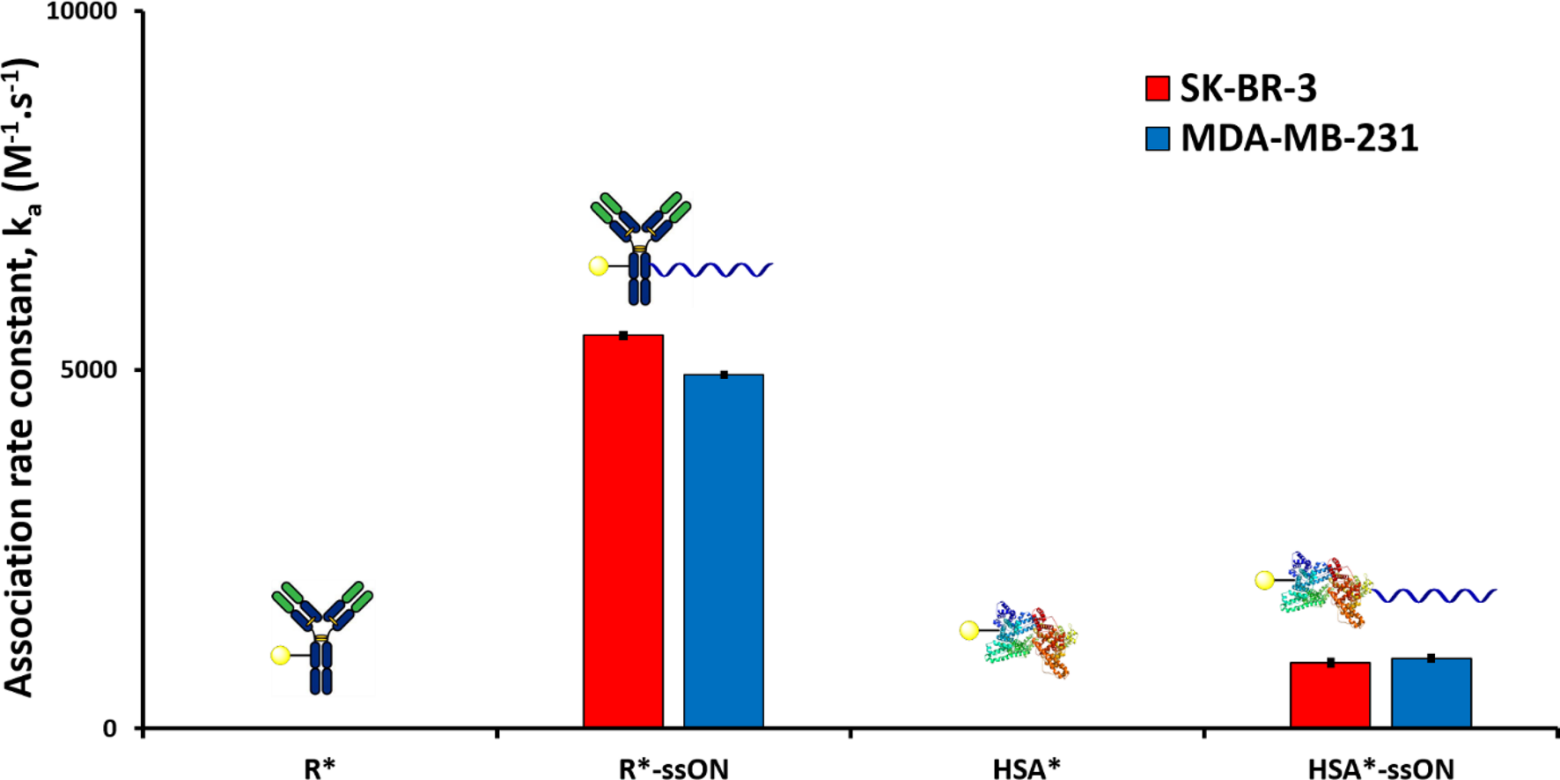
Association rate constants (k_a_) of unconjugated rituximab (R*), single stranded rituximab-ssON conjugate (R*-ssON), unconjugated HSA (HSA*), and single stranded HSA-ssON conjugate (HSA*-ssON) on SK-BR-3 (HER2^+^ cell line, red bars) and MDA-MB-231 (HER2^−^ cell line, blue bars) measured using the time-resolved analysis of ligand binding assay (Fig. S7-10). The symbol * indicates fluorescein-labelling. Error bars indicate the standard error of the fitted parameter k_a_.

To validate this observation, we conjugated the same single-stranded 37mer ON to rituximab (R), an antibody targeting B-lymphocyte antigen CD20 (an antigen that is expressed by neither SK-BR-3 nor MDA-MB-231), and to HSA (Fig. 3).

As expected, both non-conjugated rituximab (R*) and HSA (HSA*) showed no interaction with either cell line. On the other hand, the corresponding ON conjugated R*-ssON and HSA*-ssON were shown to interact to an undeniable extent with both cell lines (Fig. 3), which is consistent with our previous observation that the conjugation of an ON to a protein induces an interaction with cells that is mediated by the ON moiety and not the protein. Interestingly, this effect was more pronounced with R*-ssON than HSA*-ssON, indicating that different proteins might be impacted differently by ON conjugation.

To gain further evidence, we performed a competition assay, where MDA-MB-231 cells, supposedly interacting with T*-ssON via non-specific interactions, were pre-incubated with a 100-fold excess of free 37mer ssON, relative to T*-ssON (Fig. S17).

As expected, we found that the addition of free ssON to the medium almost completely prevented the further association of T*-ssON with MDA-MB-231, suggesting a shielding effect from the free ssON.

**Figure 4:**
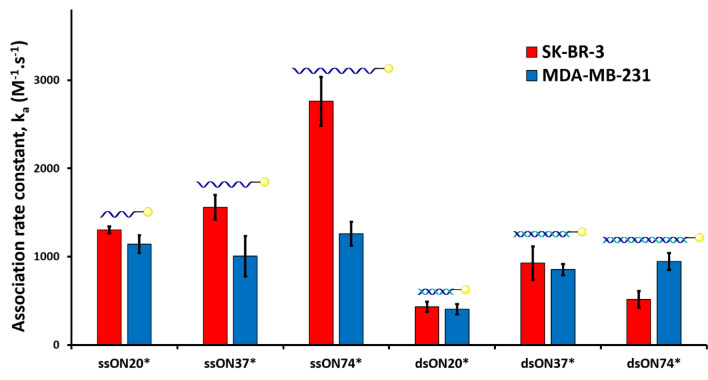
Association rate constants (k_a_) of free 20mer, 37mer, and 74mer, single and double-stranded ONs (respectively ssON20*, ssON37*, ssON74*, and dsON20*, dsON37*, dsON74*) on SK-BR-3 (HER2^+^ cell line, red bars) and MDA-MB-231 (HER2^−^ cell line, blue bars) measured using the time-resolved analysis of ligand binding assay (Fig. S11-16). The symbol * indicates fluorescein-labelling. Error bars indicate the standard error of the fitted parameter k_a_.

We then evaluated the influence of the ONs’ length and hybridization on the association rate constant by comparing single and double stranded forms of non-coding and non-structured 20mers, 37mers and 74mers. In all cases, weak interactions with both cell lines were observed, with consistently higher values for single-stranded species (Fig. 4 and SI).

**Figure 5:**
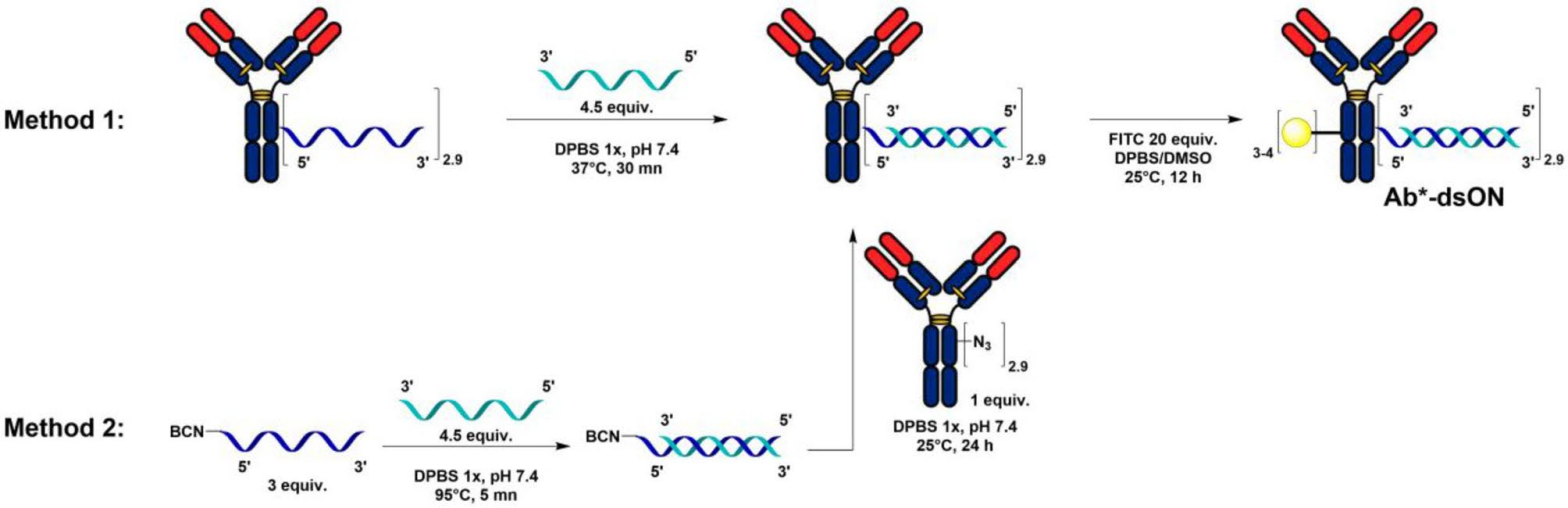
General scheme for the synthesis and labelling of Ab*-dsON conjugates.

**Figure 6:**
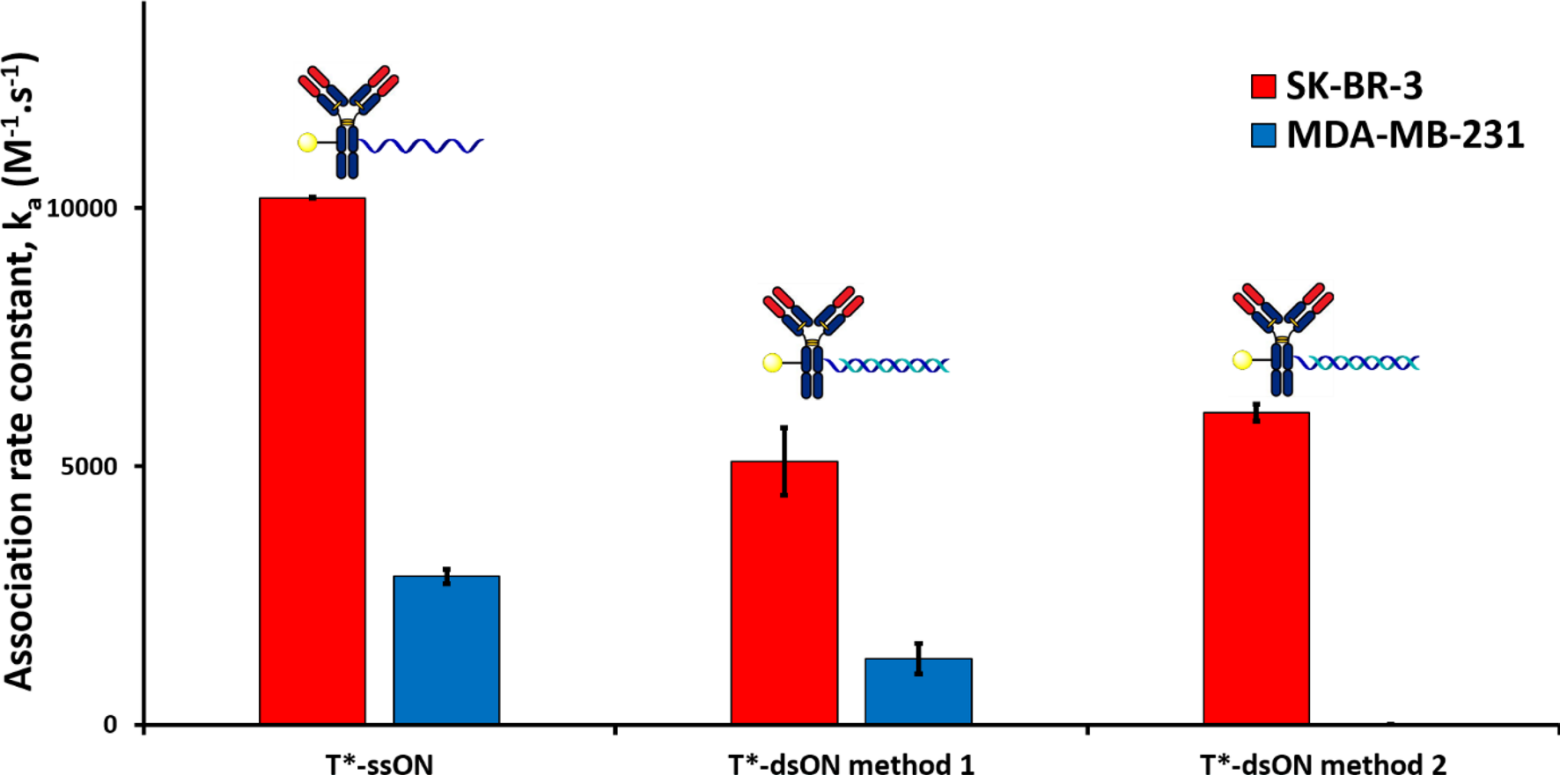
Association rate constants (k_a_) of single and double stranded trastuzumab-ssON conjugate (T*-ssON and T*-dsON, respectively), and free single and double stranded ONs (ssON* and dsON*, respectively) on SK-BR-3 (HER2^+^ cell line, red bars) and MDA-MB-231 (HER2^−^ cell line, blue bars) measured using the time-resolved analysis of ligand binding assay (Fig. S4-6). The symbol * indicates fluorescein-labelling. Regarding the T*-dsON conjugates modes of preparation, method 1 corresponds to the hybridization of the complementary ON strand with the T-ssON conjugate, while method 2 corresponds to the conjugation of a hybridized dsON to trastuzumab (see Fig. 5). Error bars indicate the standard error of the fitted parameter k_a_.

In the context of drug delivery applications of AOCs^2,14–20^, the ON component is often hybridized with its complementary strand in the form of double-stranded ONs (dsONs). As this could lead to weaker interactions with cell membranes, based on the results in Fig. 4, we set to prepare a dsON version of our trastuzumab conjugate (T*-dsON) in order to evaluate the effect of such structural change. A first method to prepare this conjugate consists in the hybridization of a complementary ssON (cON) to the previously synthesized T-ssON. This is typically done by a brief incubation at 37 °C of the two partners, in order to prevent degradation of the antibody (method 1, Fig. 5). This approach is mostly employed for non-covalent conjugation^21^ between two molecules that had been separately conjugated with complementary ssONs^2,20,22,23^. The validity of this approach is supported by the many examples of immunoassays (e.g. immuno-PCR^24,25^, proximity extension assay^26,27^, protein arrays^28^), which rely upon hybridization steps that proceed under similar conditions. A second method consists in hybridizing the two strands under classical conditions (i.e. by incubation at 95 °C) prior to the bioconjugation step (method 2, Fig. 5). This method has been reported for the synthesis of antibody-siRNA conjugates from commercial chemically-modified duplexed siRNAs^14,16^. We produced T*-dsON conjugates with identical DoC values of 2.9 (determined by SDS-PAGE gel analysis; see Fig. S1) using both methods and compared their interaction rate constants to those of T*-ssON on both cell lines (Fig. 6).

For the T*-dsON conjugates prepared following the first method, switching from single to double-stranded ONs gave comparable non-specific interactions, and also resulted in a slight decrease in k_a_, notably on the HER2^+^ SK-BR-3 cell line. Interestingly, the conjugate prepared following the second method had similar k_a_ with SK-BR-3 cells but did not display non-specific interactions with MDA-MB-231 cells.

In the polymerase chain reaction (PCR), full hybridization of ONs is highly dependent on temperature, and the initial denaturation step is typically performed at 94 °C^29^. Incubation at 90–95 °C is thus commonly used for the hybridization of complementary ssONs into dsONs, as in the case of method 2 (Fig. 5). Performing the hybridization at lower temperature is useful when working with protein-ssON conjugates, since they are prone to undergo thermal denaturation, but it might not be sufficient to reach full hybridization (method 1, Fig. 5). This could account for the interaction profile of the T*-dsON conjugate prepared by method 1 which is halfway between that of the fully hybridized T*-dsON, prepared by method 2, and that of T*-ssON.

The association of naked ONs with cells surface has now been studied for more than 50 years, with many cell-surface proteins proposed as receptors^4^. It has been documented that various types of ONs might bind to different receptors. As an example, toll-like receptors (TLRs), involved in the innate immune response, possess the ability to bind DNA molecules containing CpG motifs, dsRNAs as well as ssRNAs^30^. Cell-surface receptors of the scavenger receptors family, such as stabilin, have been reported to bind and internalize phosphorothioate-modified oligodeoxynucleotides^31^, despite some conflicting results having been published^32^. Furthermore, proteins of the systemic RNA interference defective protein 1 (SID1) transmembrane family, such as SIDT-1 and SIDT-2, have been shown to facilitate the uptake of ssRNA and dsRNA but not of ssDNA and dsDNA^33–35^.

Our results show that, when conjugated to an antibody, ONs are not simple linkers nor spectator payloads. Based on the present work and this body of literature, our group is currently investigating further the mechanism by which these interactions between AOCs and cell membranes operate at the molecular level and can be controlled.

## Conclusion

As AOCs are developing into powerful tools in various applications^1^, investigations to get a better understanding of their interactions with cell surfaces appear to be stimulating a renewed interest^30^. Our previous^2^ and present results show that ONs are a particular payload that may display weak but consistent interactions with cell surfaces, which can impact the binding properties of antibodies upon conjugation. We demonstrate that both the nature of the ON, single strand *vs* double strand, as well as the method used to prepare the dsON AOC have a clear impact on the non-specific interaction of the resulting conjugates. This phenomenon is likely to disturb the in vitro and in vivo behavior of AOCs and influence their fate beyond what can be extrapolated from the knowledge of classical protein conjugates. As such, it appears that both ON structure and preparation method should be taken into consideration when developing antibody-oligonucleotide conjugates for imaging, detection or therapeutic application.

## Materials and methods

All reagents were obtained from commercial sources and used without prior purifications. Amino-modified (5AmMC12) oligonucleotides were purchased from IDT. Protein-oligonucleotide conjugates were purified by gel filtration using ÄKTA Pure System (isocratic elution with DPBS 1x, pH 7.5, 0.5 mL/mn, column: Superdex 200 Increase 10/300 GL). The oligonucleotide species were purified using a Shimadzu HPLC system (pumps: LC 20-AD, detector: SPD 20-A, autosampler: SIL 20-A) using a XTerra MS C18 5 μM 4.6 × 150 mm column (Waters), with a flow rate of 1 mL/mn (Mobile phase: **A** triethylammonium acetate 50 mM in water, **B** triethylammonium acetate 50 mM in acetonitrile).

### Conjugates synthesis

#### Oligonucleotide functionalization

BCN-PEG_6_-PFP (**1**) was synthesized as previously described^7^. In a 2 mL Eppendorf tube, 5′-amino-modified oligonucleotide (1 equiv., 50 µL, 1 mM in water) was combined with **1** (20 equiv., 50 µL, 20 mM in DMSO) and NaHCO_3_ (100 equiv., 5 µL, 1 M in water). The mixture was incubated at 25 °C overnight. The mixture was then diluted with water to a final volume of 300 µL and added with acetone (900 µL) and LiClO_4_ (20 µL, 3 M in water) in order to precipitate the oligonucleotide species. The sample was then centrifuged (15,000 G, 8 mn) and the supernatant was discarded. The precipitate was dissolved with water (300 µL) to repeat the precipitation and centrifugation procedure a second time.

#### Oligonucleotide purification

The previously obtained precipitate was then dissolved with water (100 µL) and purified by HPLC (detection at 260 nm, mobile phase gradient A/B 9:1 to 6:4 in 30 mn). After lyophilization, the ON conjugate was dissolved in DPBS (1x, pH 7.4) and analyzed by absorption spectrophotometry (measured at 260 nm using a Nanodrop) to calculate the solution’s concentration using Beer-Lambert’s law.

#### Protein azido-functionalization

4-azidobenzoyl fluoride (ABF, **2**) was synthetized as previously described^7^.** 2** (3 equiv., 10 mM in DMSO) was added to a solution of protein (1 equiv., 5 mg/mL, 100 µL in DPBS 1x, pH 7.4) and the reaction mixture was incubated at 25 °C for 30 min. The excess of reagents was then removed by gel filtration chromatography using Bio-spin P-30 Columns (Bio-Rad, Hercules, U.S.A.) pre-equilibrated with DPBS (1x, pH 7.4) to give a solution of protein-azide conjugates, which was used in the following step.

#### Protein-oligonucleotide conjugates synthesis

The previously obtained 5′-BCN-modified oligonucleotide (3 equiv., 0.5–1 mM in DPBS 1x) and 10 µL of DPBS 10 × were added to a solution of the protein-azide conjugate (1 equiv., 5.0 mg/mL, in 100 µL DPBS 1x, pH 7.4). The mixture was purged with argon and incubated for 24 h at 25 °C. The conjugates were purified by gel filtration chromatography using AKTA Pure System (isocratic elution with DPBS (1x, pH 7.4), 0.5 mL/min) to yield the protein-oligonucleotide conjugates.

#### Fluorescein labelling

5′-fluorescein labelled (56-FAM) oligonucleotides were purchased from IDT.

Proteins were concentrated to 1–5 mg/mL on micro-concentrators (Vivaspin, 50 and 10 kD cutoff, for antibodies and HSA, respectively, Sartorius, Gottingen, Germany), and added with 20 equiv. of FITC (10 mM in DMSO). The mixture was then incubated at 25 °C overnight. The excess of FITC was then removed by gel filtration chromatography using Bio-spin P-30 Columns (Bio-Rad, Hercules, U.S.A.) pre-equilibrated with DPBS 1x (pH 7.5) to give a solution of FITC-labelled proteins.

#### Double stranded oligonucleotides and antibody-oligonucleotide conjugates synthesis

Free oligonucleotides were hybridized by stirring a solution of the complementary strands at an equimolar ratio (100 µM) in DPBS 1 × at 95 °C for 5 mn, and then allowing the solution to come back to room temperature. The hybridized species were then purified from the non-hybridized ones using AKTA Pure System (isocratic elution with DPBS (1x, pH 7.4), 0.5 mL/min).

The double stranded AOCs were prepared by two methods (Fig. 5):

##### Method 1

T-ssON conjugate was mixed with an excess (4.5 equiv.) of complementary strand in DPBS 1x (pH 7.4), incubated at 37 °C for 30 min, and purified by gel filtration chromatography using AKTA Pure System (isocratic elution with DPBS (1x, pH 7.4), 0.5 mL/min) to yield T-dsON.

##### Method 2

5′-BCN-modified oligonucleotide (3 equiv., 2.06 nmole, 400 µM in DPBS 1x), and its complementary strand (4.5 equiv., 3.09 nmole, 1 mM in DPBS 1x) were mixed and stirred at 95 °C for 5 min, and then allowed to slowly return to room temperature. The mixture was then added with 3 µL of DPBS 10x, and then with the above-described azido-modified trastuzumab (1 equiv., 100 µg, 4.3 mg/mL). After incubation at 25 °C for 24 h, the formed T-dsON37 conjugate was purified using AKTA Pure System (isocratic elution with DPBS (1x, pH 7.4), 0.5 mL/min).

### Conjugates’ characterization

#### Protein-oligonucleotide conjugates concentration determination

The concentration of a protein in a given solution can usually be determined by measuring its absorption at 280 nm, and using Beer-Lambert’s law. ON’s absorbance at 280 nm thus makes it impossible to determine the concentration of protein-oligonucleotide conjugates through absorption spectrophotometry measurement.

Protein-oligonucleotide conjugates’ concentration was then determined using Pierce BCA protein assay kit (ThermoFisher ref 23225), following the manufacturer’s protocol. This method allows quantification of the protein moiety’s concentration, regardless of the presence of conjugated ONs. Concentrations were used to calculate the yields of conjugation (see Table S1).

#### Protein-oligonucleotide conjugates DoC distribution determination by SDS PAGE

SDS-PAGE was performed on 4–20% Mini-PROTEAN TGX Gel (Bio-Rad ref 4561094) following the manufacturer’s procedure. Proteins, or protein-oligonucleotide conjugates (24 µL, 0.2 mg/mL in DPBS 1x) were added with 8 µL of non-reducing Laemmli SDS sample buffer (Alfa Aesar), and heated at 95 °C for 5 mn. 10 µL of the resulting solutions were deposited, and the gel was run at constant voltage (200 V) for 35 mn using TRIS 0.25 M—Glycine 1.92 M—SDS 1% as a running buffer. Coomassie Blue staining was performed using Instant*Blue* solution, prior to visualization on GeneGenius bio-imaging system (Syngene).

The lines’ intensities were determined using the Image Studio Lite 5.2 software (LI-COR Biosciences), and the DoC of each conjugate was calculated using the following formula (Eq. 1):

1 $$DoC = \frac{{\sum\limits_{k} {{\text{k }} \times {\text{ I}}({\text{DoCk}}} )}}{{\sum\limits_{k} {{\text{I}}({\text{DoCk}}} )}}$$

where I(DoC_k_) is the line’s intensity of the conjugate with k conjugated oligonucleotides per antibody.

Additionally, we analyzed the deglycosylated azido-modified trastuzumab intermediate by native mass spectrometry (see figure S2). As observed in a previous work^7^, the mean DoC values obtained by integration of SDS-PAGE gel bands of the Ab-ssON conjugate (2.9) and native MS of the azido-modified intermediate (2.8) were closely correlated.

#### Proteins and protein-oligonucleotide conjugate degree of labelling (DoL) determination

After FITC-labelling, the fluorescein concentration of each protein and protein-ON conjugate was measured by absorption spectrophotometry using NanoDrop’s “proteins and labels” mode.

The DoL of each compound was calculated using Eq. (2) (see Table S1).

2 $$DoL = \frac{fluorescein\, concentration\, (M)}{Protein\, concentration\, (M)}$$

For fluorescein-labelled proteins, the protein concentration was determined by absorption at 280 nm, while for fluorescein-labelled protein-ON conjugates, it was determined by BCA assay (see above).

### Binding assays

#### Cell culture

Human breast adenocarcinoma cells SK-BR-3 (ATCC HTB-30) and MDA-MB-231 (ATCC HTB-26)) were grown in Dulbecco’s Modified Eagle’s Medium (DMEM) containing 4.5 g/L glucose (Sigma, St Louis, MO, USA). The medium was supplemented with 10% fetal bovine serum (Perbio, Brebieres, France), 2 mM L-Glutamine, 100 U/mL Penicillin and 100 µg/mL Streptomycin (Sigma). Cells were maintained in a 5% CO2 humidified atmosphere at 37 °C.

SK-BR-3 cells overexpress HER2 protein, and MDA-MB-231 cells are used as negative controls.

The day before the experiment on Ligand Tracer Green (Ridgeview Instruments), cells were seeded as 600 μL droplets with 8 × 105 cells/mL near the edge in 87 mm cell culture treated dishes (Greiner, Frickenhausen, Germany) and incubated at 37 °C overnight. Two droplets were prepared with SK-BR-3, one with MDA-MB-231, and one was left as a background reference (plastic).

Prior to kinetic measurements, the medium was carefully removed and 3 mL of fresh complete medium was added to the dish.

#### Time-resolved analysis of ligand binding assays

We measured the interactions of fluorescein-labelled proteins and oligonucleotides with living cells in real-time using LigandTracer Green (Ridgeview intruments).

The Petri dish on which cells were grown (see above) was placed on the inclined rotating support of the instrument, with the Blue/Green detector placed on its upper part.

First, a baseline signal was collected for 30 min. The fluorescein-labelled compound was then added in two steps at increasing concentrations (10 and 30 nM). Inclination of the Petri dish allows for the addition of the fluorescein-labelled compounds outside of the detection area. For each rotation of the Petri dish, the signal from the three areas containing cells (two spots for SK-BR-3, one for MDA-MB-231) and a background reference area (plastic) is recorded. Measurements last 30 s each, with 5 s in between each of them to allow the medium to sit in the lower part of the Petri dish. This results in three background-subtracted real-time binding curves, which represent the binding of the fluorescein-labelled compound to each cell-containing area.

For each concentration the incubation was performed until a sufficient curvature was obtained for the subsequent extraction of kinetic parameters. Dissociation of the ligand was recorded after replacing the incubation solution with 3 ml of fresh medium.

Signals from cell and reference areas are recorded during every rotation, resulting in a background-subtracted binding curve. Binding traces were analyzed with the evaluation software TraceDrawer 1.8.1 (Ridgeview Instruments) in order to determine k_a_ according to the Langmuir, or “one-to-one”, binding model.

#### Competition assay

After collection of the baseline signal, 100 equiv. of unlabelled ssON37 (relative to the total added amount of T*-ssON) were added to the medium. After 30 mn, the time-resolved analysis of T*-ssON binding was performed as previously described (Fig. S17).

## Supplementary Information

Supplementary Information
