# Supplementary material for: Non-specific interactions of antibody-oligonucleotide conjugates with living cells
Source: Sci Rep. 2021 Mar 15;11:5881. doi: 10.1038/s41598-021-85352-w (PMC7961061; doi:10.1038/s41598-021-85352-w)
Supplement: Supplementary file 1 — Supplementary Information [file 41598_2021_85352_MOESM1_ESM.docx]

**Supporting Information**

**Non-specific interactions of antibody-oligonucleotide conjugates with living cells**

Victor Lehot, Isabelle Kuhn, Marc Nothisen, Stéphane Erb, Sergii Kolodych, Sarah Cianférani, Guilhem Chaubet and Alain Wagner^*^

Bio-Functional Chemistry (UMR 7199)

# LaBex MEDALIS, UMR 7199

# Faculté de Pharmacie

# Université de Strasbourg

# 74 route du Rhin

# CS 60024

# 67401 ILLKIRCH CEDEX

# Email: alwag@unistra.fr

# Oligonucleotides’ sequences design:

The oligonucleotide sequences were designed in such a way that the GC content was around 50% and that they did not adopt any stable secondary structure. GC content was calculated using *eq. 1*. The stability of the potential secondary structures of the oligonucleotides was predicted using IDT’s UNAFold tool (eu.idtdna.com/unafold/Home/Index).

$GC content = \frac{G + C}{G + C + A + T}$ (*eq. 1*)

# Oligonucleotide sequences:

## Sequences used for protein-oligonucleotide conjugates:

**ssON:** 5’-AmMC12-AA GAT ACG AAT TCG GGT GTT CTG CTG GTA GTG GTC GG-3’

**dsON:** ssON hybridized with 5’-CCG ACC ACT ACC AGC AGA ACA CCC GAA TTC GTA TCT T-3’

## Sequences used for free oligonucleotides:

**ssON20*:** 5’-56FAM-CG TAC AGT GGA GCA GAT TAC-3’

**dsON20*:** ssON20* hybridized with complementary strand (5’- GT AAT CTG CTC CAC TGT ACG -3’);
Tm = 52.7 °C

**ssON37*:** 5’-56FAM-CCG ACC ACT ACC AGC AGA ACA CCC GAA TTC GTA TCT T-3’

**dsON37*:** ssON37* hybridized with complementary strand (5’-AA GAT ACG AAT TCG GGT GTT CTG CTG GTA GTG GTC GG-3’); Tm = 66.4 °C

**ssON74*:** 5’-56FAM-CT TGA TCA AAG TCA GCG TCA GTC TCT TGA GAT CTC GGT AGT GCA GTG GTC ATA ACG TAC AGT GGA GCA GAT TAC-3’

**dsON74*:** ssON74* hybridized with complementary strand (5’-GTA ATC TGC TCC ACT GTA CGT TAT GAC CAC TGC ACT ACC GAG ATC TCA AGA GAC TGA CGC TGA CTT TGA TCA AG-3’); Tm = 69.7 °C


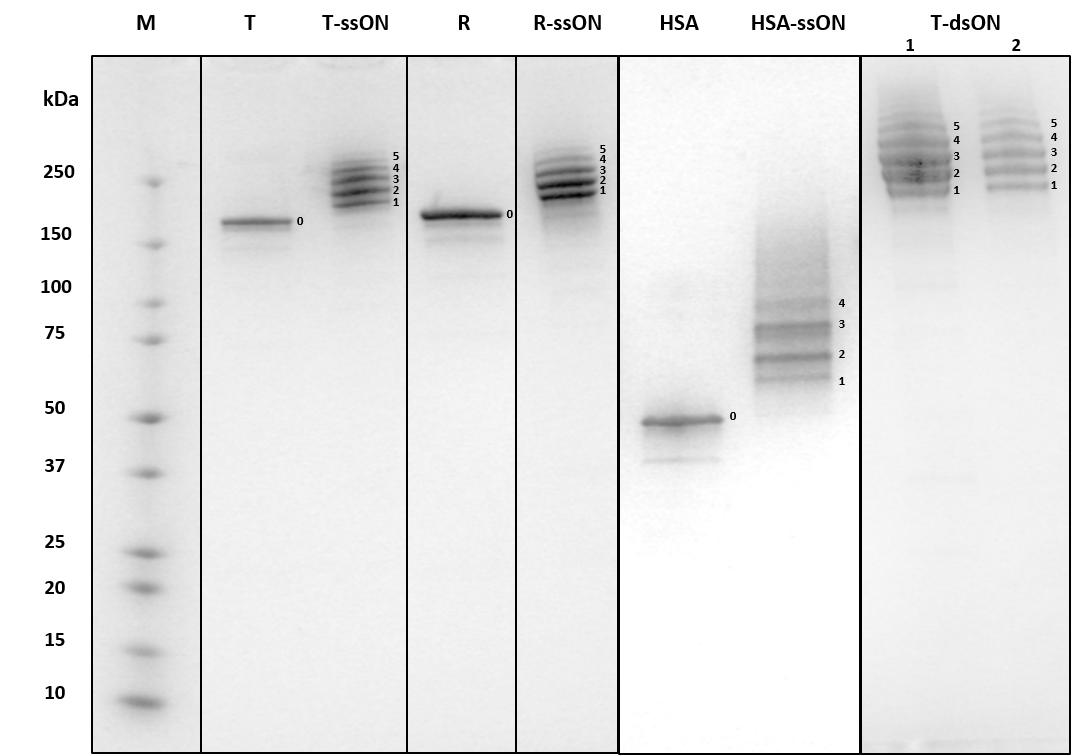


Figure S1. DoC distribution observed by SDS-PAGE (4-15%). Next to each lane the DoC of the corresponding conjugate is indicated by a number. T-dsON 1 and 2 correspond to T-dsON conjugates prepared using methods 1 and 2 (see material and methods section) respectively. Gels were cropped from three gels and reorganized to facilitate interpretation. Full original gels are available at the end of the ESI (see Fig. S18-20).


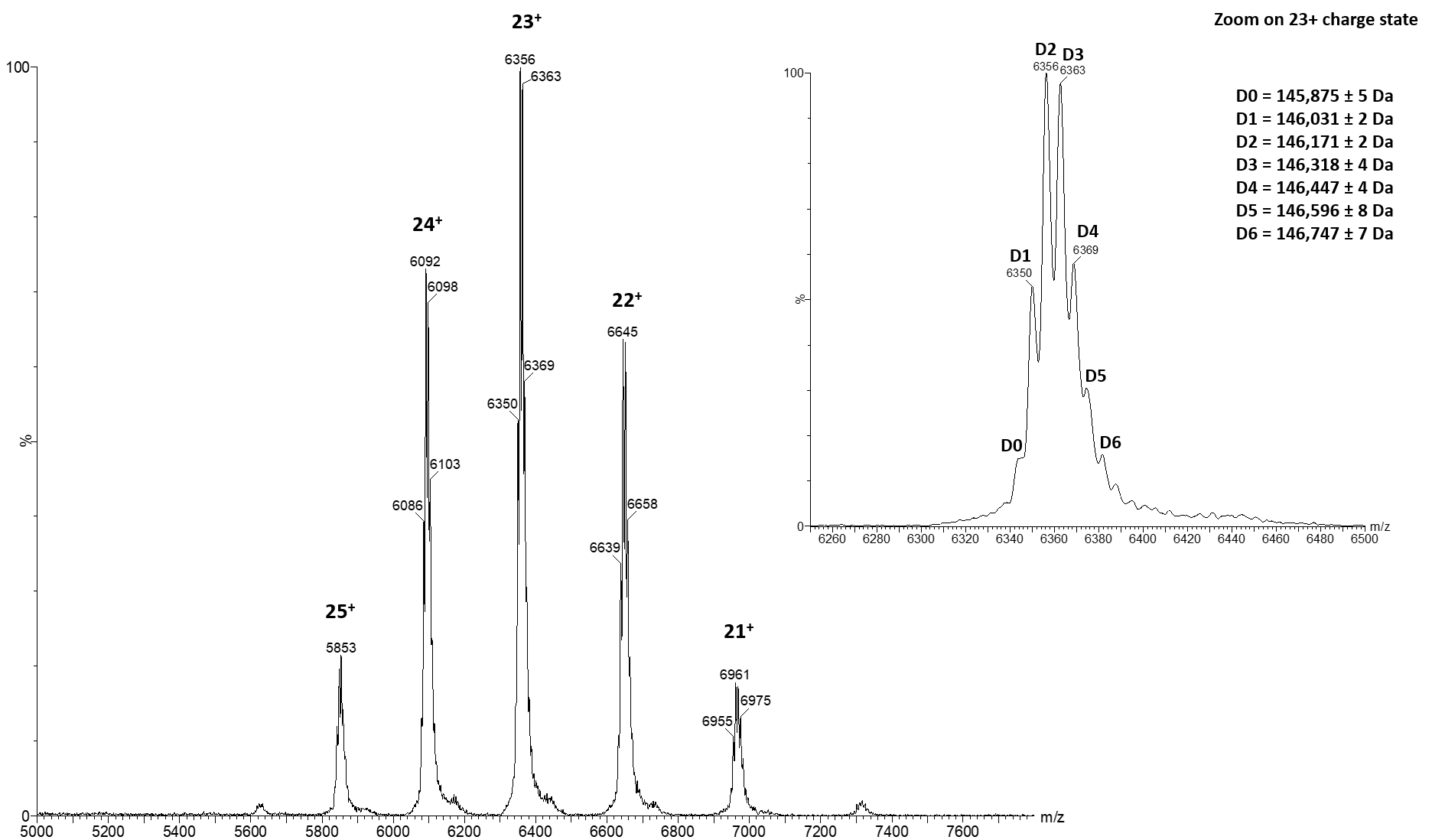


Figure S2. Mass spectrum (left) and zoom on 23+ charge state (top right) of the azido-modified Trastuzumab intermediate.

| Compound | MW | Yield | DoL | DoC |
| --- | --- | --- | --- | --- |
| T* | 145532 | - | 1.7 | - |
| T*-ssON | 179752 | 57% | 4.9 | 2.9 |
| ssON* | 11800 | - | 1 | - |
| R* | 143860 | - | 1.4 | - |
| R*-ssON | 178080 | 57% | 1.1 | 2.9 |
| HSA* | 66437 | - | 3.3 | - |
| HSA*-ssON | 93577 | 33% | 2.3 | 2.3 |
| T*-dsON | 213972 | 57% (method 1) 31 % (method 2) | 3.4 3.5 | 2.9 2.9 |
| dsON* | 23600 | - | 1 | - |
| ssON20* | 6300 | - | 1 | - |
| dsON20* | 12600 | - | 1 | - |
| ssON74* | 23000 | - | 1 | - |
| dsON74* | 46000 | - | 1 | - |

Table S1. Characteristics of the fluorescein-labelled compounds. The Molecular weights (MW) of the conjugates are calculated using: MW_conjugate_ = MW_protein_ + DoC x MW_oligonucleotide_. The yield of conjugation is defined as the amount of protein-oligonucleotide conjugate obtained after oligonucleotide conjugation (determined by BCA assay, see material and methods section) divided by the initial amount of protein engaged in the reaction. The degree of conjugation (DoC) is defined as the mean number of oligonucleotide per protein (determined by SDS-PAGE gel, see material and methods section). The degree of labelling (DoL) is defined as the mean number of fluorescein molecules per protein (determined by absorption spectrophotometry and BCA assay, see material and methods section).


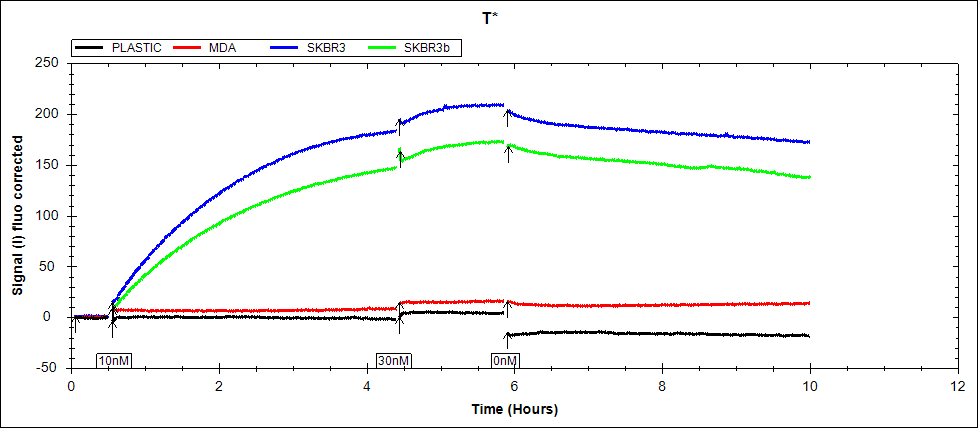


Figure S3. Time-resolved analysis of T* binding. The first set of arrows represent the beginning of the experiment. The next two consecutive sets of arrow indicate the first and second addition of fluorescein-labelled compound, and the last set represents the medium refreshment. Black, red, blue and green lines represent the signals obtained from the plastic, MDA-MB-231 cells spot, and the two SK-BR-3 cells spots, respectively.


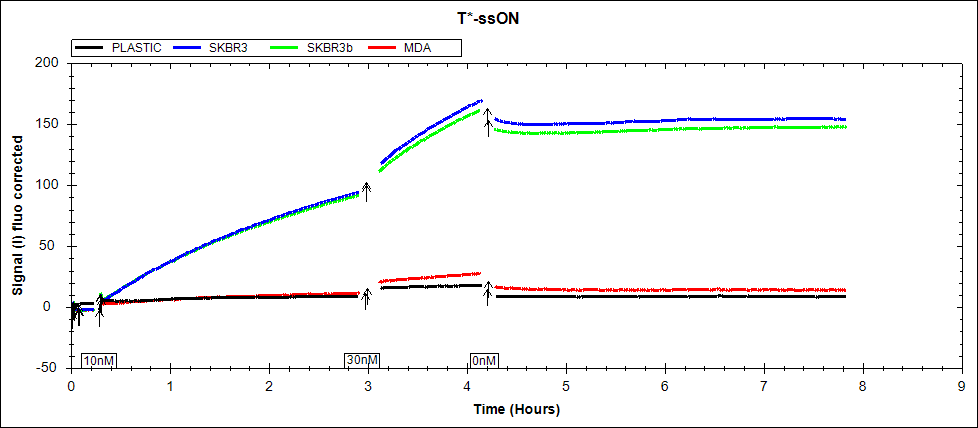


Figure S4. Time-resolved analysis of T*-ssON binding. The first set of arrows represent the beginning of the experiment. The next two consecutive sets of arrow indicate the first and second addition of fluorescein-labelled compound, and the last set represents the medium refreshment. Black, red, blue and green lines represent the signals obtained from the plastic, MDA-MB-231 cells spot, and the two SK-BR-3 cells spots, respectively.


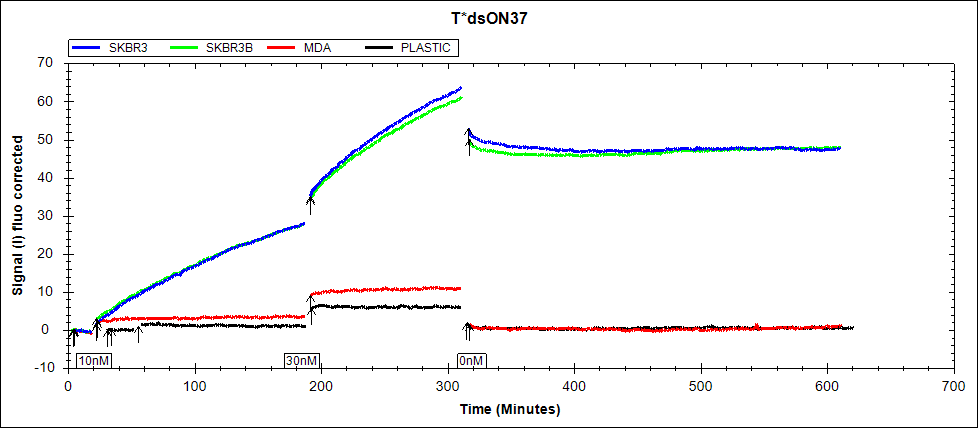


Figure S5. Time-resolved analysis of T*-dsON binding prepared by the method 1 (see experimental section). The first set of arrows represent the beginning of the experiment. The next two consecutive sets of arrow indicate the first and second addition of fluorescein-labelled compound, and the last set represents the medium refreshment. Black, red, blue and green lines represent the signals obtained from the plastic, MDA-MB-231 cells spot, and the two SK-BR-3 cells spots, respectively.


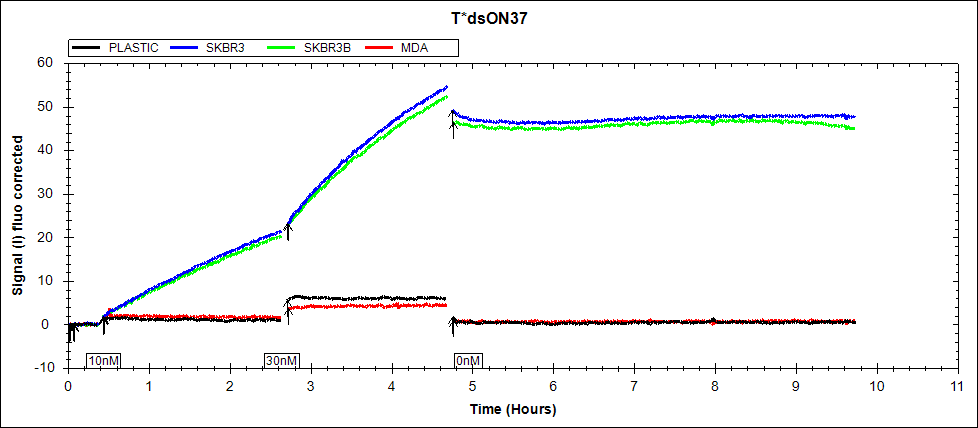


Figure S6. Time-resolved analysis of T*-dsON binding prepared by the method 2 (see experimental section). The first set of arrows represent the beginning of the experiment. The next two consecutive sets of arrow indicate the first and second addition of fluorescein-labelled compound, and the last set represents the medium refreshment. Black, red, blue and green lines represent the signals obtained from the plastic, MDA-MB-231 cells spot, and the two SK-BR-3 cells spots, respectively.


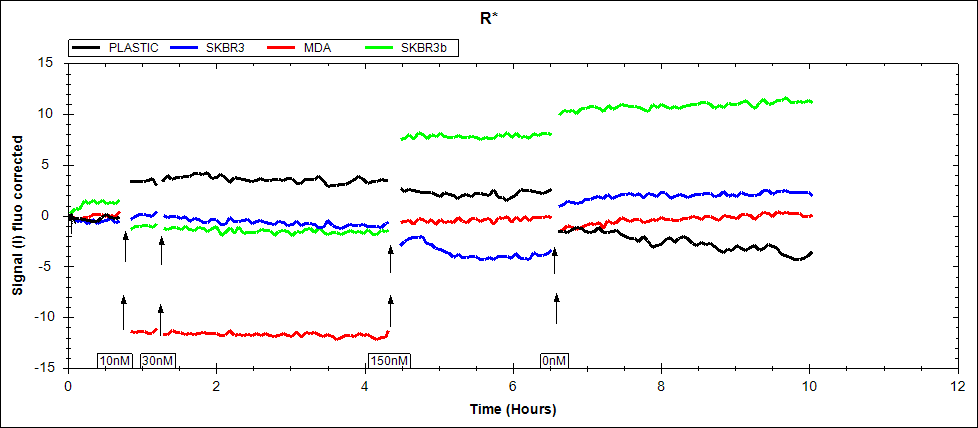


Figure S7. Time-resolved analysis of R* binding. The first set of arrows represent the beginning of the experiment. The next three consecutive sets of arrow indicate the first and second addition of fluorescein-labelled compound, and the last set represents the medium refreshment. Black, red, blue and green lines represent the signals obtained from the plastic, MDA-MB-231 cells spot, and the two SK-BR-3 cells spots, respectively.


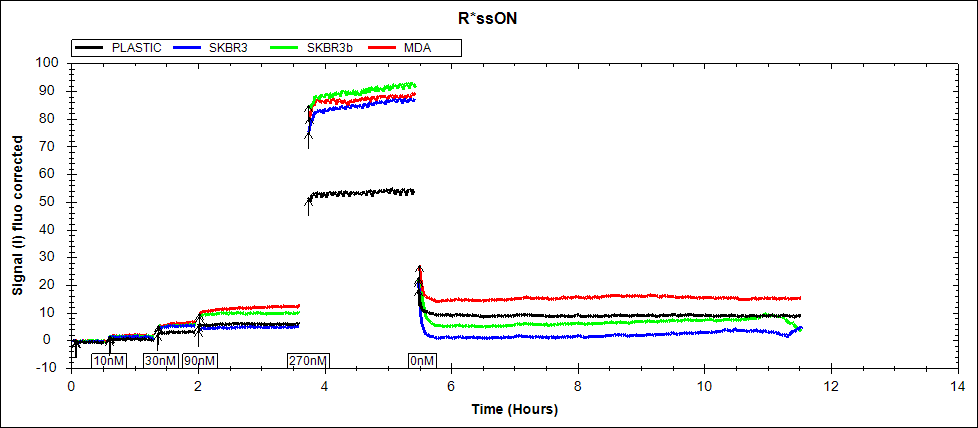


Figure S8. Time-resolved analysis of R*-ssON binding. The first set of arrows represent the beginning of the experiment. The next four consecutive sets of arrow indicate the first and second addition of fluorescein-labelled compound, and the last set represents the medium refreshment. Black, red, blue and green lines represent the signals obtained from the plastic, MDA-MB-231 cells spot, and the two SK-BR-3 cells spots, respectively.


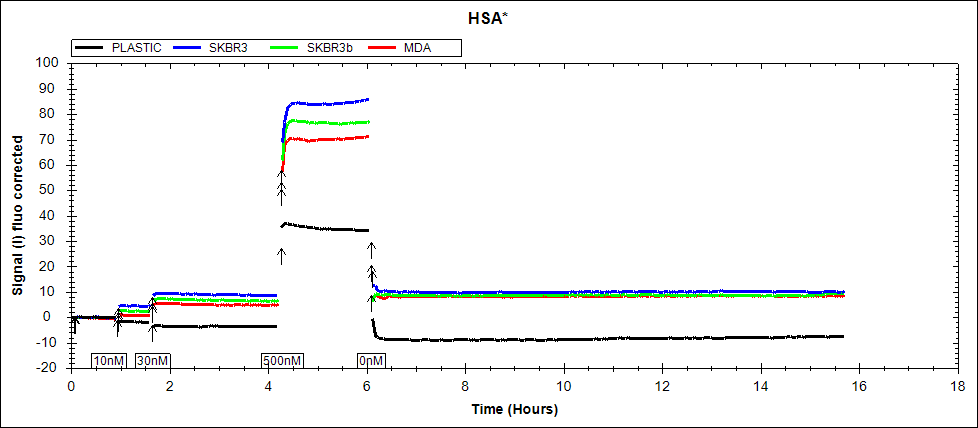


Figure S9. Time-resolved analysis of HSA* binding. The first set of arrows represent the beginning of the experiment. The next three consecutive sets of arrow indicate the first and second addition of fluorescein-labelled compound, and the last set represents the medium refreshment. Black, red, blue and green lines represent the signals obtained from the plastic, MDA-MB-231 cells spot, and the two SK-BR-3 cells spots, respectively.


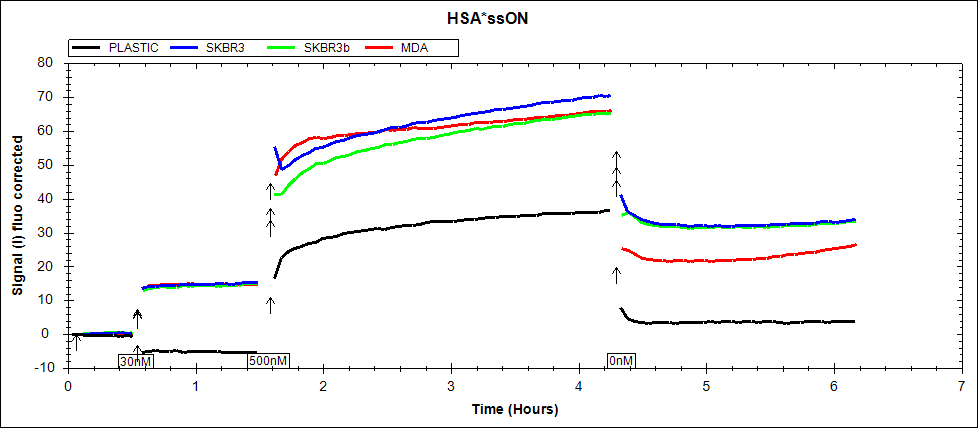


Figure S10. Time-resolved analysis of HSA*-ssON binding. The first set of arrows represent the beginning of the experiment. The next two consecutive sets of arrow indicate the first and second addition of fluorescein-labelled compound, and the last set represents the medium refreshment. Black, red, blue and green lines represent the signals obtained from the plastic, MDA-MB-231 cells spot, and the two SK-BR-3 cells spots, respectively.


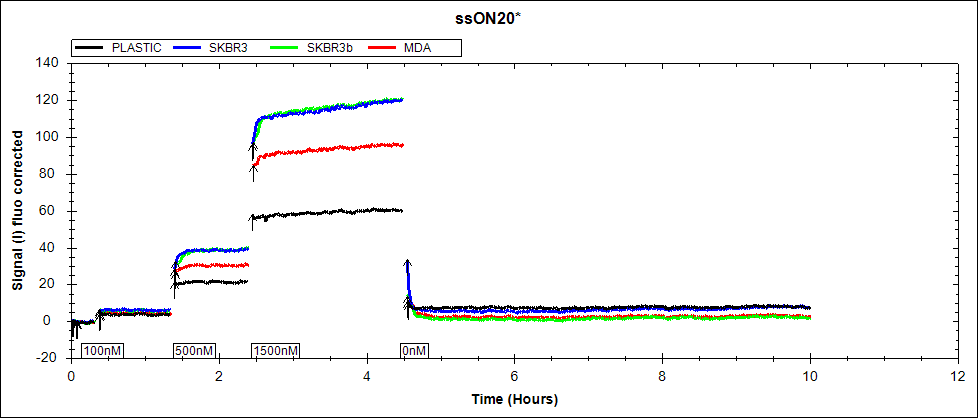


Figure S11. Time-resolved analysis of ssON20* binding. The first set of arrows represent the beginning of the experiment. The next two consecutive sets of arrow indicate the first and second addition of fluorescein-labelled compound, and the last set represents the medium refreshment. Black, red, blue and green lines represent the signals obtained from the plastic, MDA-MB-231 cells spot, and the two SK-BR-3 cells spots, respectively.


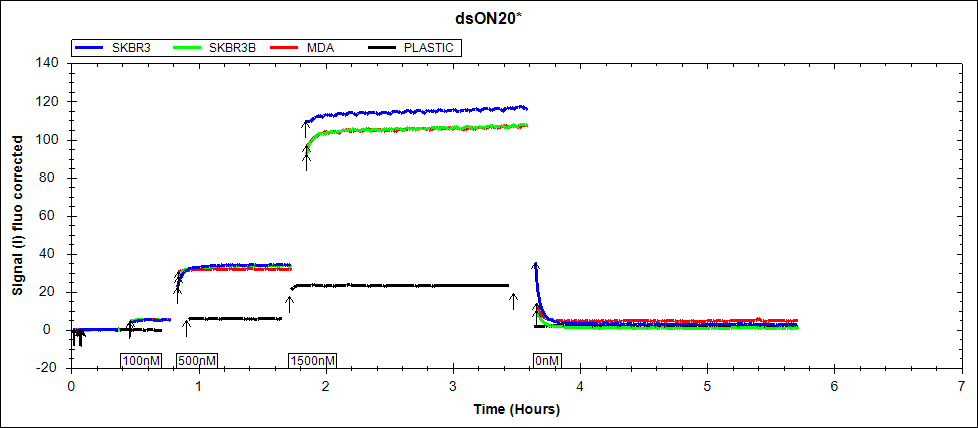


Figure S12. Time-resolved analysis of dsON20* binding. The first set of arrows represent the beginning of the experiment. The next two consecutive sets of arrow indicate the first and second addition of fluorescein-labelled compound, and the last set represents the medium refreshment. Black, red, blue and green lines represent the signals obtained from the plastic, MDA-MB-231 cells spot, and the two SK-BR-3 cells spots, respectively.


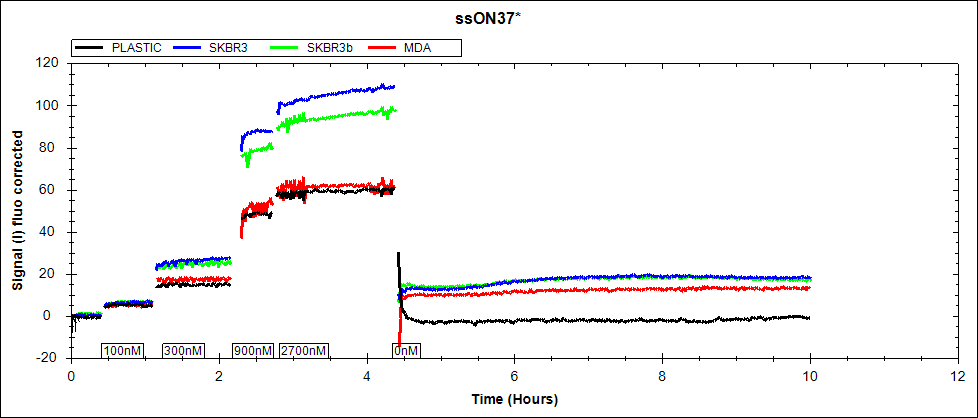


Figure S13 Time-resolved analysis of ssON37* binding. The first set of arrows represent the beginning of the experiment. The next two consecutive sets of arrow indicate the first and second addition of fluorescein-labelled compound, and the last set represents the medium refreshment. Black, red, blue and green lines represent the signals obtained from the plastic, MDA-MB-231 cells spot, and the two SK-BR-3 cells spots, respectively.


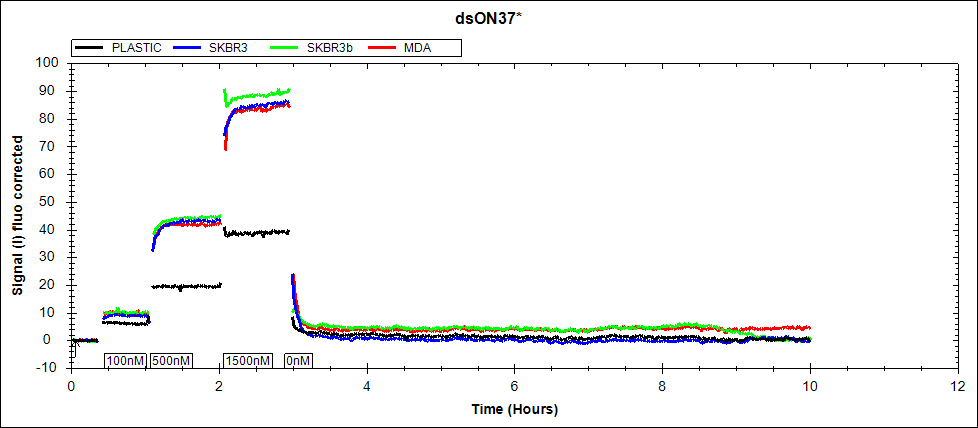


Figure S14. Time-resolved analysis of dsON37* binding. The three consecutive sets of arrow indicate the first and second addition of fluorescein-labelled compound, and medium refreshment, respectively. Black, red, blue and green lines represent the signals obtained from the plastic, MDA-MB-231 cells spot, and the two SK-BR-3 cells spots, respectively.


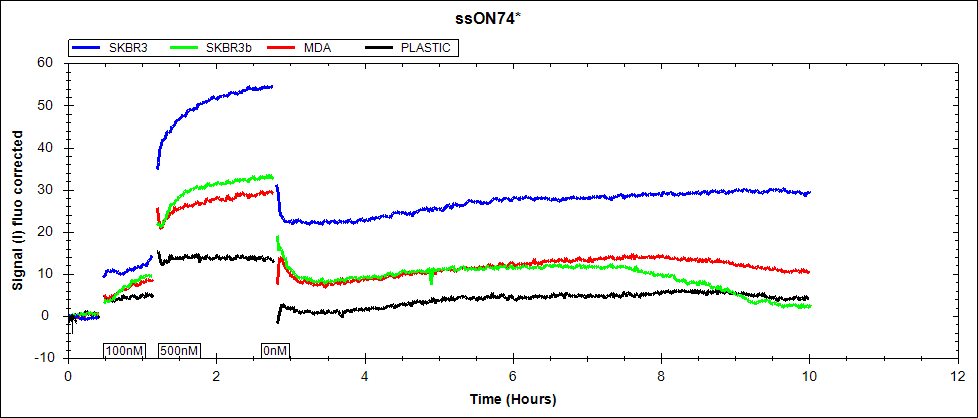


Figure S15. Time-resolved analysis of ssON74* binding. The three consecutive sets of arrow indicate the first and second addition of fluorescein-labelled compound, and medium refreshment, respectively. Black, red, blue and green lines represent the signals obtained from the plastic, MDA-MB-231 cells spot, and the two SK-BR-3 cells spots, respectively.


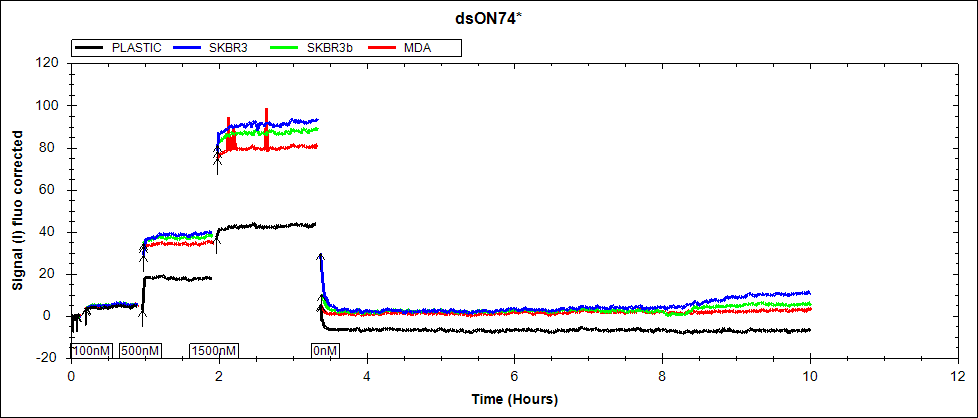


Figure S16. Time-resolved analysis of dsON74* binding. The three consecutive sets of arrow indicate the first and second addition of fluorescein-labelled compound, and medium refreshment, respectively. Black, red, blue and green lines represent the signals obtained from the plastic, MDA-MB-231 cells spot, and the two SK-BR-3 cells spots, respectively.


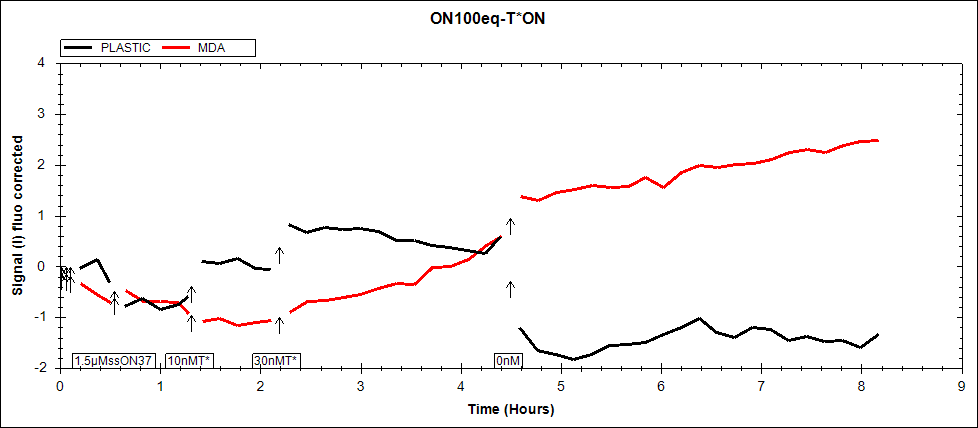


Figure S17. Time-resolved analysis of T*-ssON binding to MDA-MB-231, following incubation with 0 (left) and 100 equiv. (right) of unlabelled ssON37 (top). The three consecutive sets of arrow indicate the first and second addition of fluorescein-labelled compound, and medium refreshment, respectively (bottom). Black, and red lines represent the signals obtained from the plastic, and the MDA-MB-231 cells spot, respectively.


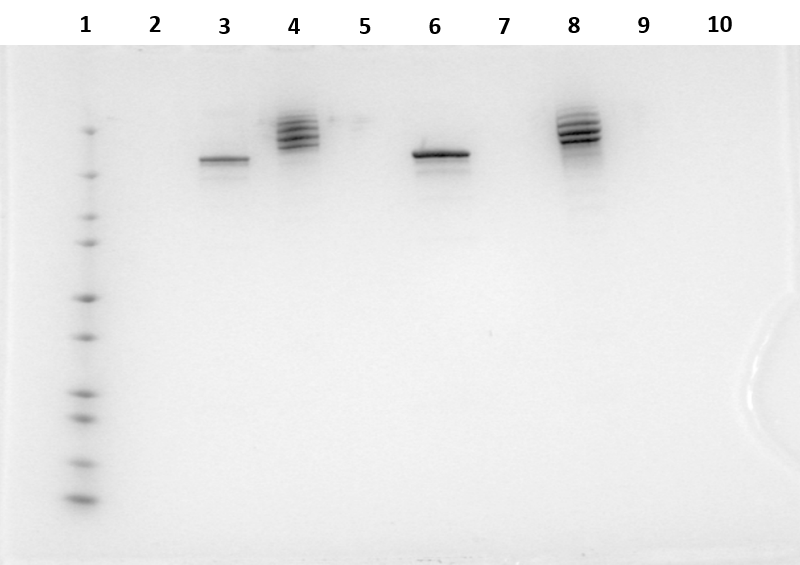


Figure S18. Full gel where lanes 1, 3, 4, 6 and 8 are protein ladder, T, T-ssON, R, and R-ssON from Fig. S1, respectively.


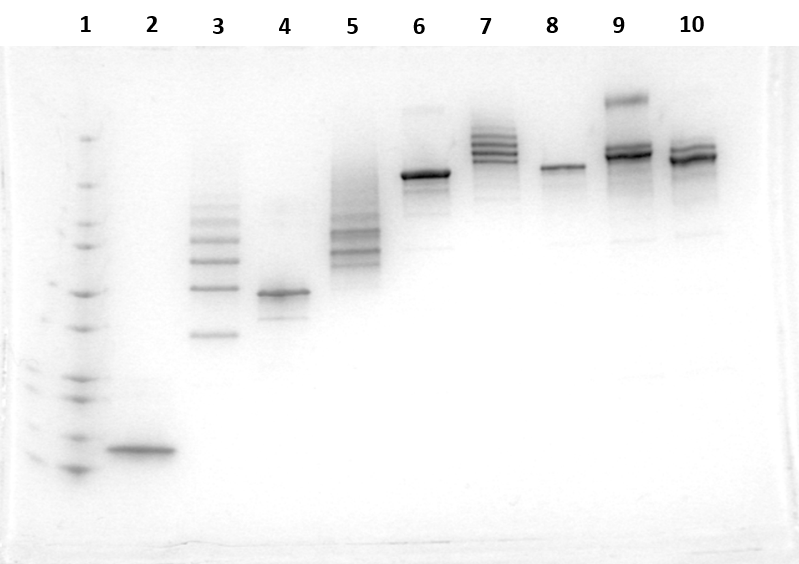


Figure S19. Full gel where lane 4 is HSA and lane 5 is HSA-ssON from Fig. S1.


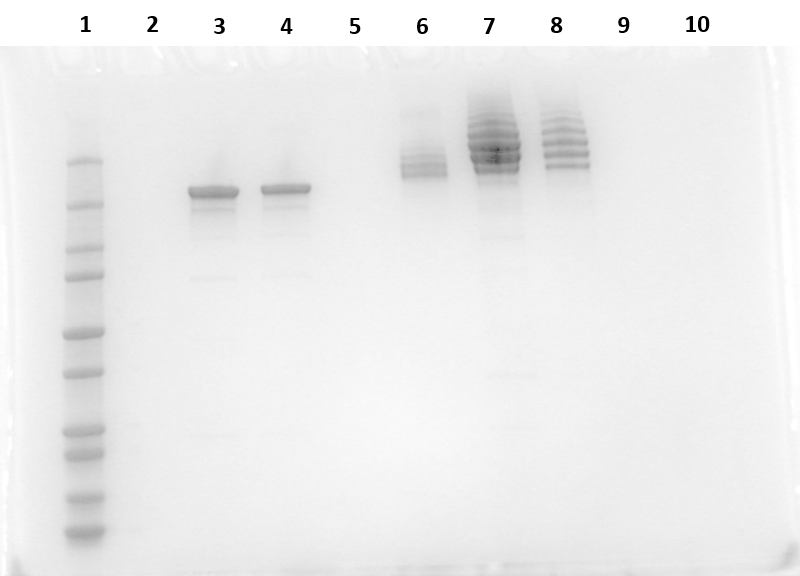


Figure S20. Full gel where lanes 7 and 8 are the T-dsON conjugates (prepared from methods 1 and 2 respectively) from Fig. S1.
